# Effect of Heat Treatment on Microstructure and Properties of 304/Q235 Composite Round Steel

**DOI:** 10.3390/ma18112497

**Published:** 2025-05-26

**Authors:** Xiexin Zheng, Yi Ding

**Affiliations:** College of Materials Science and Engineering, Nanjing Tech University, Nanjing 210009, China; zzz1111@njtech.edu.cn

**Keywords:** 304/Q235, heat treatment, carbide, fatigue property, corrosion resistance

## Abstract

During the heat treatment of stainless steel (SS)/carbon steel (CS) bimetal composites, the carbon in the CS diffuses into the SS, and carbides precipitate on the grain boundary and in the grains, affecting the microstructure and properties of the composite steel. In order to change the precipitation and distribution of the carbides seen on hot-rolled 304/Q235 after cold drawing (HR), the microstructure and properties of composite round steel were investigated by optical microscopy, SEM/EDS, and hardness, tensile, fatigue, and electrochemical tests while changing the temperature of the full annealing and aging treatments. The results showed that dispersed chromium carbide particles precipitated at the grain boundaries, and intragranular and slip lines promoted simultaneous dispersion strengthening and fine-grain strengthening and greatly improved the hardness, yield strength, tensile strength, and fatigue strength of the composite round steel. However, the increase in chromium carbide particles leads to the formation of stress concentration points and accelerates the creation of fatigue cracks, resulting in a decrease in the fatigue strength of the steel. Simultaneously, the corrosion resistance of the composite round steel samples was reduced due to the precipitation of a large amount of chromium carbide.

## 1. Introduction

Q235 steel is widely used in the production of parts and building structures because of its good overall performance and low cost; however, its corrosion resistance is poor, meaning that it cannot be used in corrosive environments [1]. Moreover, 304 austenitic stainless steel is one of the SSs most widely used in engineering [2]; while it has excellent corrosion resistance, its mechanical properties and fatigue strength are relatively poor, such that it cannot be widely used in environments where high strength is required. Bimetallic composites are prepared using advanced composite technology, which enables the tight combining of two different metallic materials to form a novel metallic structure [3,4]. This technology has received extensive attention within the realm of engineering [5,6,7]. It not only retains the excellent performance of both metals but also generates synergistic functions, making it superior to other composite-forming technologies, highly valuable to society [8], and able to meet the demands of various application scenarios [9]. In recent years, the technology used to create bimetal composites has greatly advanced due to increasing demand for these composites, with more and more bimetal profiles—such as pipes, bars, and fasteners—being produced [10,11,12]. However, there are still many problems to be solved after a composite metal has been created, such as the residual stress generated during the process, which affects the mechanical properties, fatigue resistance, and corrosion resistance of the material.

Heat treatments are often used to ensure that bimetallic composites meet different performance requirements after they are produced, meaning that selecting an appropriate heat treatment process is crucial. Tan et al. [13] found that the mechanical properties of a single metal were improved by normalizing its treatment to promote martensitic transformation and eliminate the element segregation zone. The tensile strength and impact toughness of U71Mn steel were improved after this normalizing treatment. Sarkar et al. [14] found that full annealing produced a refined and uniform structure, eliminated structural defects, and improved plasticity and toughness. Wang et al. [15] confirmed that a solution treatment fully dissolved various phases found in SS and strengthened the solid solution, thereby improving the toughness and corrosion resistance of the material. The diffusion of elements often occurs during the heat treatment of bimetals. Wang et al. [16] and Ding et al. [17] found that when two metal components interact, the elemental diffusion that occurs leads to the formation of intermetallic compounds at the composite bond. Dhib et al. [18] studied the mechanical properties and interface morphology of austenitic SS cladding plates and found that the diffusion of carbon between CS and SS changed the near-surface microstructure of the cladding plates. In SS/CS bimetallic composites, the diffusion of carbon atoms during a heat treatment produces carbon–chromium compounds along austenite grain boundaries, resulting in a decline in corrosion resistance and potentially leading to the brittle fracture of the material under the action of external forces, thus limiting the improvement in the mechanical properties of bimetallic composites [19]. On the contrary, this kind of carbon–chromium compound—which is dispersed in the grain after precipitation—can also improve hardness and wear resistance and effectively hinder dislocation during plastic deformation, thus strengthening the performance of the metal. The formation and precipitation of carbides are directly related to temperature and time. A higher temperature can accelerate the diffusion rate of chromium and carbon atoms, and a longer time allows chromium and carbon atoms to diffuse more fully. This promotes precipitation, which affects the corrosion resistance, mechanical strength [20], and fatigue strength of the metal.

When metal structures fail in practical applications, it is mainly due to brittle fracture, ductile fracture, corrosion, creep, or fatigue. Any one of these can result in immeasurable economic losses and casualties. Metal is especially prone to fatigue fracture under the action of cyclic alternating stress [21,22]. However, because there is no obvious plastic deformation before fatigue fracture in metals, it is difficult to prevent instantaneous fatigue fracture accidents. In recent years, research has focused on the fatigue properties of single types of steel, such as low-CS, and there have been a few studies on the fatigue properties of composite metal materials. Therefore, it is of practical significance to study how to improve the fatigue performance of bimetallic composites, as it could provide ideas for improving the fatigue strength of composites, broaden the areas in which bimetallic composites can be used, and increase their service life.

In this study, Q235 CS and 304 SS were hot-rolled before being combined into 304/Q235 through cold drawing (HR). The experimental samples were prepared by changing the temperature of their full annealing (FA) and aging treatment (AT). The precipitation of carbides after different heat treatments and its effects on the microstructure, hardness, strength, fatigue life, and corrosion resistance of 304/Q235 were studied using metallographic analysis, energy-dispersive spectroscopy (EDS), hardness tests, tensile tests, rotating bending fatigue tests, field-emission scanning electron microscopy (SEM), and electrochemical tests.

## 2. Experimental Procedure

### 2.1. Preparation and Heat Treatment

**Figure 1:**
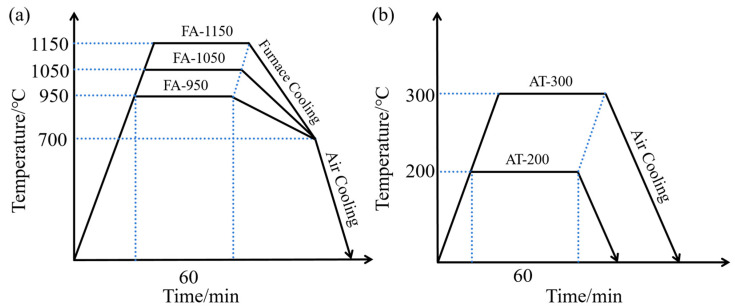
Diagram of heat treatment process: (**a**) full annealing; (**b**) aging treatment.

In this study, 304/Q235 composite round steel was prepared using the hot rolling method, and plastic deformation was induced by the downward pressure of the roller, which metallurgically bonded the two metals through local fusion and occlusion. The specific steps were as follows: a 304 SS tube with an outer diameter of 18 mm and a wall thickness of 2 mm was sheathed on Q235 CS round bar with a diameter of 14 mm before being kept at 1200 °C for 60 min. The composite round steel underwent six rounds of continuous rolling immediately after being removed from the heat. An HR with a length of 120 mm and diameter of 12 mm was obtained after the cold drawing treatment; its chemical composition is shown in Table 1. We used FA and AT as our heat treatment processes. The specific steps of the FA were as follows: the holding temperature was set at 950 °C (FA-950), 1050 °C (FA-1050), or 1150 °C (FA-1150); the HRs were held at the predetermined temperature for 60 min and then cooled to 700 °C in the furnace before being cooled to room temperature in air. The specific steps of the AT were as follows: we set the temperature at 200 °C or 300 °C and kept FA-950 at different predetermined temperatures for 60 min, before removing it from the furnace and cooling it to room temperature. The heat treatment process and nomenclature of composite round steels are shown in Figure 1.

### 2.2. Characterization of Microstructure and Properties of Composites

**Figure 2:**
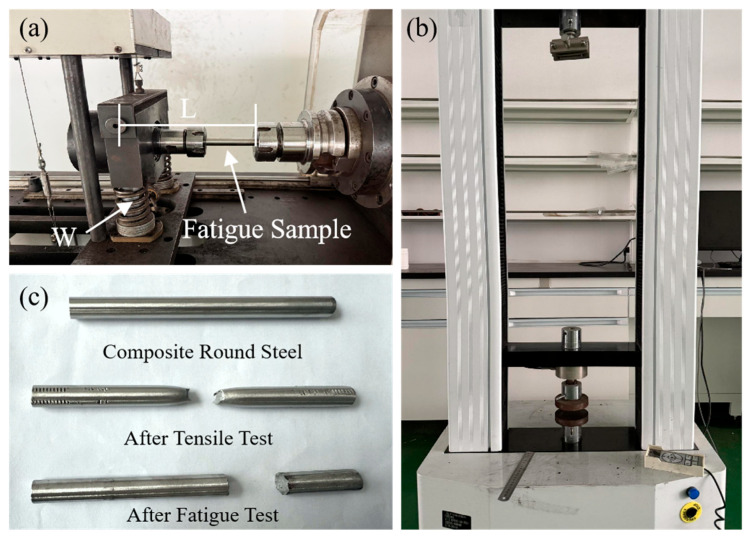
Experimental machine and samples: (**a**) rotating bending fatigue machine; (**b**) universal testing machine; (**c**) samples before and after testing.

The composite round steel was cut into 10 mm long metallographic samples by wire cutting. The CS layer is etched using 4 wt.% nitric acid alcohol after polishing. The microstructure of the CS was observed through an optical microscope, and the microstructure of the SS was observed after electrolysis had been performed 100 times in 10 wt.% oxalic acid solution (voltage of 7 V and time of 10 s). SEM and EDS were used to study the diffusion and distribution of different elements after the heat treatment. The hardness of the CS core and SS cladding was measured using a Vickers hardness tester with a load of 100 gf, loading time of 20 s, and unloading time of 10 s. The interface between the two metals in the composite round steel was used as the reference for measurements made in the cross-section direction: 15 points were measured within 700 μm of the interface for the SS layer; 8 points were evenly selected from within 400 μm of the other side of the interface for the CS layer; and all the test data were processed by the three-point average method to ensure the test’s accuracy. Uniaxial tensile tests were performed on a universal testing machine (Figure 2b), and the tensile fracture morphology of the samples were observed using SEM. The fatigue test was carried out on an XM-1000 cantilever beam rotating bending fatigue testing machine (Figure 2a). The load W/N used in the fatigue test is shown in Formula (1). Here, d/mm is the diameter of the composite round steel, σ/MPa is the maximum surface bending stress, and L/mm is the distance from the maximum surface bending stress section to the origin. The speed was set to 3000 r/min.

W = πd^3^σ/32L (1)

The morphology of the resultant fatigue fractures was observed using SEM. The traditional three-electrode structure was used in our electrochemical experiments; the saturated calomel electrode was used as the reference electrode, the platinum electrode as the counter electrode, and the sample as the working electrode. The electrochemical impedance spectroscopy (EIS) and potentiodynamic polarization curves were measured in 3.5 wt.% NaCl solution (PH = 7) after the open circuit’s potential became stable, and the double-loop potentiodynamic reactivation (DL-EPR) was performed in H_2_SO_4_ (0.5 mol/L) + KSCN (0. 01 mol/L) solution at a room temperature of 20 °C.

## 3. Results and Discussion

### 3.1. Microstructure and Mechanical Properties

**Figure 3:**
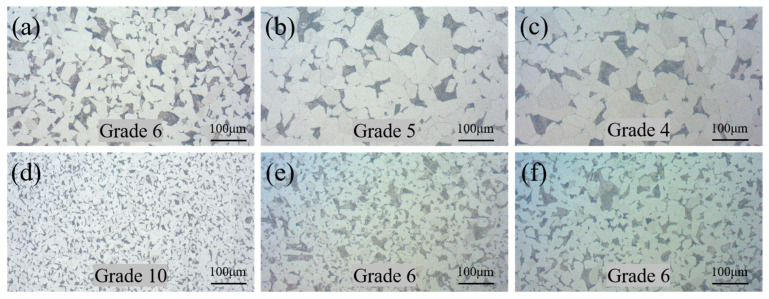
Microstructure of CS in composite round steel: (**a**) FA-950; (**b**) FA-1050; (**c**) FA-1150; (**d**) HR; (**e**) AT-200; (**f**) AT-300.

Figure 3 shows the microstructure of the CSs after different heat treatments. It can be seen that the microstructure of the CS in each sample is composed of ferrite (light-colored area) and pearlite (lamellar structure). Figure 3d shows that the grain size of the HR sample is the finest, at 10.0. With the increase in the full annealing temperature from 950 °C to 1150 °C, the austenitic grain boundary migrates further, and the grain size of the austenite gradually increases, which leads to a decrease in the grain size of the ferrite–pearlite structure after cooling. At the same time, after aging treatments at either 200 °C and 300 °C, the grain size of the CS does not change significantly and is about 6.0, which is equal to the grain size of the FA-950 sample that was not aged.

**Figure 4:**
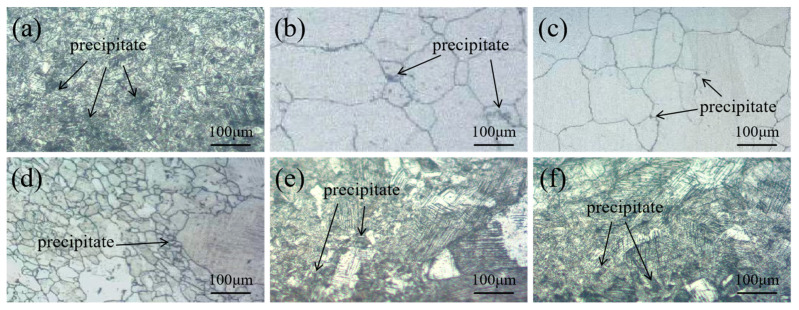
Microstructure of SS in composite round steel: (**a**) FA-950; (**b**) FA-1050; (**c**) FA-1150; (**d**) HR; (**e**) AT-200; (**f**) AT-300.

Figure 4 shows the microstructure of the SS in the samples after different heat treatments. Figure 4a shows that significant carbon diffusion occurs from the CS to SS side of FA-950. Additionally, the second-phase particles that are not completely dissolved significantly weaken the migration of the austenite grain boundaries and inhibit the growth of austenite grains through the pinning effect. Figure 4b,c show that the precipitates are completely dissolved, and the austenite grains become coarse through interface migration when annealed at 1050 °C and 1150 °C, temperatures which are higher than the solution temperature of 304 SS. Some precipitates preferentially precipitate at the grain boundary and some precipitate in the grains during cooling. As can be seen from Figure 4d, abnormal coarsening of the austenite grains occurs in the SS of HR due to the high rolling temperature and rapid rolling involved in the process. It can be observed from Figure 4e,f that there are many slip lines and twins in the austenite grain, which is the result of a large amount of plastic deformation. We also found that there are carburized layers in the SS cladding, while a large number of carbides are precipitated from grain boundaries and intragranular and slip lines.

**Figure 5:**
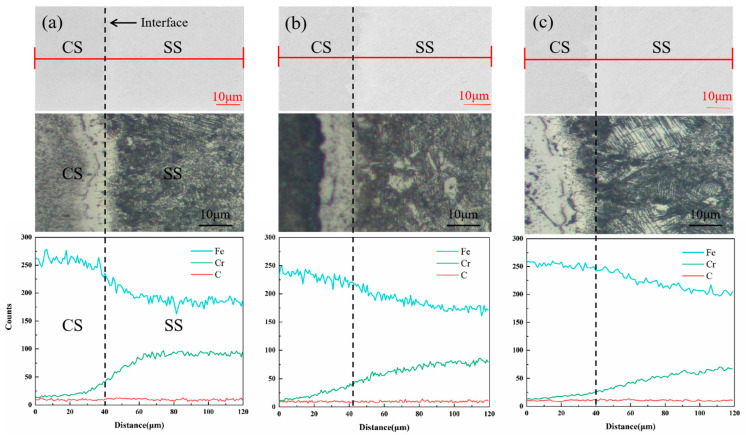
Line scanning diagrams and metallographic diagrams of interface of composite round steel: (**a**) FA-950; (**b**) AT-200; (**c**) AT-300.

**Figure 6:**
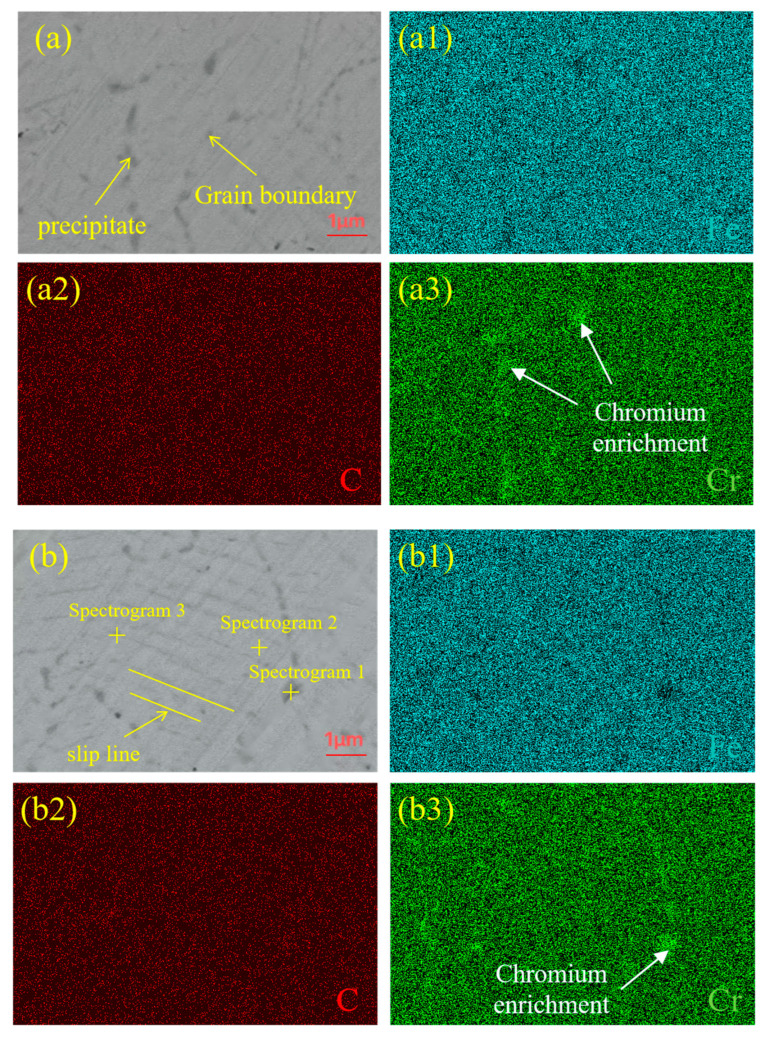
Grain boundary of SS cladding of FA-950. (**a**) SEM images: (**a1**) Fe; (**a2**) C; (**a3**) Cr. (**b**) SEM images and point scanning positions: (**b1**) Fe; (**b2**) C; (**b3**) Cr.

EDS was used to perform elemental analyses at the interface of the FA-950, AT-200, and AT-300 samples, which contain more precipitates. The line scanning results and corresponding metallographic diagrams are shown in Figure 5. It can be observed that Cr and Fe are clearly diffused at the interface of the two metals due to the difference in elemental content between the CS core and SS cladding. Although the C is less diffused than Cr and Fe [23], the diffusion of C from the CS side to the SS side can still be observed at the interface, and the formation of a carburized layer is visible on the SS side. In order to further study the diffusion and distribution of these elements, the FA-950 sample was mapped and scanned, and the results are shown in Figure 6. It can be seen that the austenite has a very fine structure and there is less Fe at the grain boundary, while Cr is enriched at the grain boundary. This is closely related to the intergranular sensitization caused by carbide precipitation. Additionally, there is a small amount of Cr enrichment near the slip line induced by plastic deformation, indicating that the dislocation channel promotes the short-range diffusion of Cr atoms, which is consistent with the typical distribution of chromium-rich carbides at the grain boundary [24]. In order to determine the composition of the precipitates, the elements present were analyzed by EDS point scanning; the results, shown in Figure 6b, reveal that the precipitates are mainly Cr, Fe, and C. The composition and mass fractions of these elements are shown in Table 2. Since carbide particles (Cr_23_C_6_) are more likely to be formed at 700 °C—which is within the intergranular sensitization temperature range of SS [25] and consistent with the austenitic grain boundary sensitization mechanism [26]—the second-phase particles that are precipitated are chromium carbide (Cr_23_C_6_). At the same time, the highest concentration of Cr is seen at the grain boundary, while the lowest is seen in the grains. The concentration of Cr on the slip line is higher than that in the grains, which indicates that a small amount of chromium carbide also precipitates on the slip line, aligning with the classical theory that carbide preferentially nucleates in high-defect regions (grain boundaries; dislocation lines).

**Figure 7:**
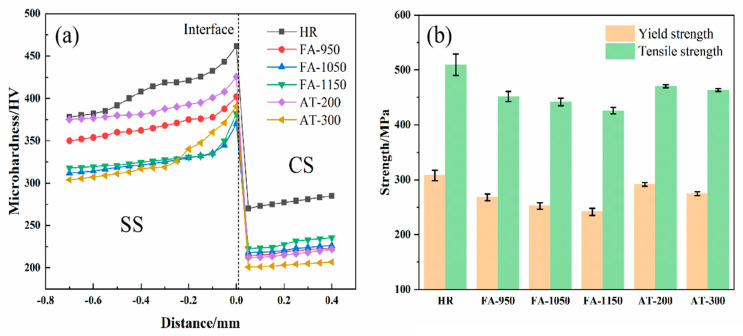
Mechanical properties of composite round steel: (**a**) hardness curve and measurement positions; (**b**) yield strength and tensile strength.

The microhardness and strength of the composite round steel samples are shown in Figure 7. Due to the existence of carbon diffusion and the limited degree of carbon diffusion in some of the samples, the SS near the bonding layer displayed the maximum hardness. Due to its cold drawing and lack of further heat treatment, the HR sample retains the cold deformation strengthening of its SS, and its microhardness is significantly higher than that of the other samples (its maximum hardness is 461.7 HV); it also has an excellent yield strength (307 MPa) and tensile strength (511 MPa). The SS of the FA-950, FA-1050, and FA-1150 samples loses its cold deformation strengthening, and its hardness is affected by the chromium carbide that formed. At the same time, FA-1050 and FA-1150 are fully austenitized, and their austenite grains are coarsened at excessively high temperatures, which leads to decreases in their hardness and grain boundary area, weakening the pinning effect, which hinders the movement of the grain boundary and causes a decrease in strength. The grain boundary acts as an obstacle to the movement of dislocations. Fine grains lead to an increase in the density of the grain boundary, meaning that dislocations become stuck at the grain boundary, such that more external force is needed to initiate plastic deformation. Therefore, the maximum hardness of FA-950, which contains the most carbide precipitates, after its full annealing treatment is 402.2 HV, while its yield strength and tensile strength are 270 MPa and 453 MPa, respectively. FA-1150, which contains the fewest carbide precipitates, has the highest hardness of 370.1 HV, and the lowest yield strength and tensile strength of all samples (244.7 MPa and 426.1 MPa, respectively), indicating that carbide contributes greatly to the hardness and strength of composite round steel. However, AT-200 is aged at a low temperature, which leads to a low diffusion rate for carbon and the slow growth of precipitates. This makes it easy to form high-density and ultra-fine carbides, which significantly improve the hardness (the maximum hardness is 425.6 HV), yield strength (291.7 MPa), and tensile strength (470.3 MPa) of this sample. With the increase in aging temperature, the precipitation of the carbides increases gradually, weakening the effect of dispersion strengthening and resulting in a decrease in the hardness and strength of AT-300.

**Figure 8:**
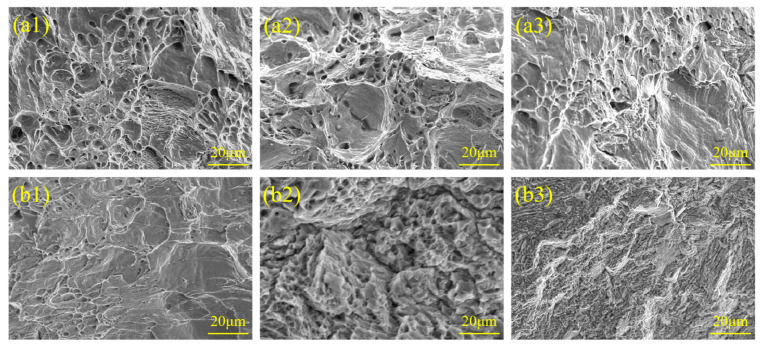
Microscopic morphology of tensile fractures: (**a1**–**a3**) AT-200; (**b1**–**b3**) AT-300.

The tensile fracture morphology of AT-200 and AT-300 was analyzed, and the results are shown in Figure 8. Figure 8(a1) reveals that the fracture morphology of AT-200 consists of half dimples and half cleavage planes. The large number of carbides precipitated on the SS side become nucleation points for the micropores, and with the accumulation of dislocation slip under tensile stress, these micropores gradually grow and polymerize, finally forming dimples. During the 200 °C aging treatment, the decarburized layer may be embrittled due to the low-temperature brittleness of ferrite and the fact that the mechanical properties of the decarburized layer are very different from those of the normal structure (pearlite) found in the core. The ratio of the migration velocity of screw dislocation to that of edge dislocation is the main factor affecting the ductile–brittle transition of the material. Edge dislocation migrates rapidly at low temperatures, while screw dislocation migrates relatively slowly. This difference in migration rates leads to a reduction in the toughness of the material. When subjected to dynamic loads or impacts, the stress concentration at the interface may promote the propagation of cracks and cause brittle fractures. It can be seen from Figure 8(b1–b3) that the tensile fracture morphology of AT-300 primarily indicates brittleness in the cleavage plane. There are small holes and secondary cracks in the SS, accompanied by radial river patterns. Short and curved rivers with few tributaries are the mark of quasi-cleavage fractures, which are somewhere between cleavage fractures and ductile fracture. A large number of carbides form along the grain boundaries or dislocation lines in the SS precipitate, creating stress concentration points. Cracks nucleate at the interface between the carbide and the matrix, and plastic deformation is hindered when these cracks propagate, such that the fracture is quasi-cleavage. In summary, both AT-200 and AT-300 produced low-temperature temper embrittlement.

### 3.2. Fatigue Properties

**Figure 9:**
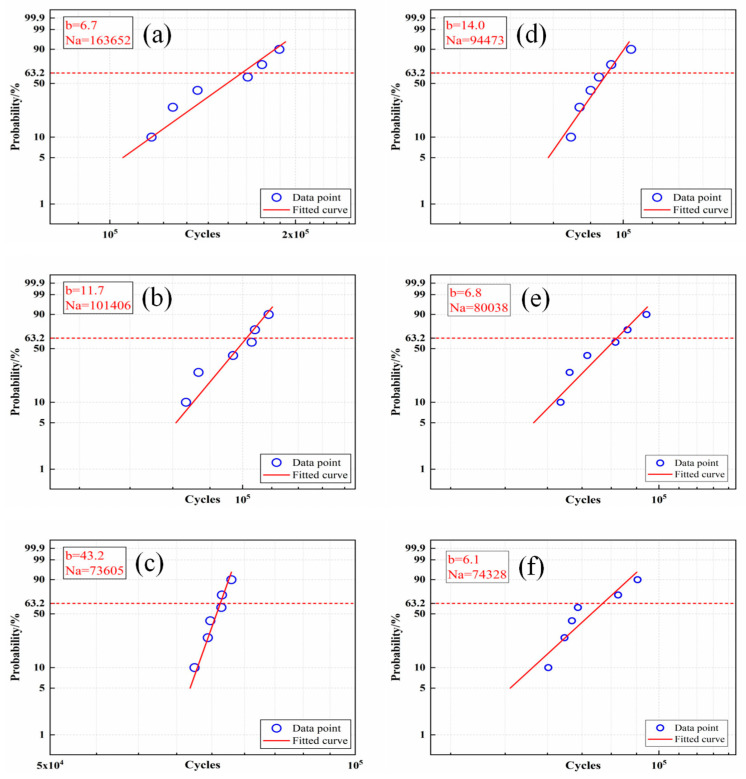
Weibull analysis diagram: (**a**) FA-950; (**b**) FA-1050; (**c**) FA-1150; (**d**) HR; (**e**) AT-200; (**f**) AT-300.

It can be seen from the data in Table 3 that, after the full annealing treatment, FA-950 lasted for the longest number of cycles under 360 MPa bending stress, averaging 152,397 cycles. FA-1150 withstood the lowest number of cycles, 72,676 on average. After further aging treatment, the fatigue life of AT-200 and AT-300 was lower than that of FA-950. A Weibull analysis was performed on the fatigue data in Table 3, and the results are shown in Figure 9. The Weibull slope parameter b and the characteristic life parameter Na were obtained at a failure probability of 63.2%. This is the same rule as that used to simply calculate the average value of the fatigue data, but it better verifies the accuracy of the experimental data. From this, it can be observed that FA-950 withstood the largest number of characteristic fatigue cycles (i.e., 163,652) under 360 MPa of bending stress, which is significantly higher than that of other samples. 

Further analysis shows that the SS of the HR sample does not play a role in dispersion strengthening because of the abnormal growth of austenite grains during hot rolling, leading to mixed-size grains. This uneven structure leads to an uneven stress distribution, meaning that the carbon cannot fully diffuse into the composite during rapid hot rolling, which affects the precipitation of carbides. Cold deformation strengthening does not play a role in improving the fatigue life of these samples. The SS of FA-950 contains a large number of carbides that are dispersed in its grains or even on the slip line, which is greatly strengthened by carbon dispersion. At the same time, the SS is harder and contains more austenite grain boundaries, which inhibits the propagation of fatigue cracks. The passivation of the crack tip will slow the propagation of fatigue cracks. Moreover, refined grains can make plastic deformation occur more uniformly, which reduces the concentration of stress and slows down the generation of cracks. As seen in FA-1050 and FA-1150, with the increase in full annealing temperature, the austenite’s grain size is coarsened and the length of the grain boundary is greatly reduced, which weakens the hindering of the grain boundary’s movement and the effect of dispersion strengthening, accelerating the initiation and propagation rate of cracks and subsequently reducing fatigue strength. Although more carbides are precipitated from AT-200 and AT-300 after an aging treatment at lower temperatures, their fatigue strength does not increase but decreases with the occurrence of temper brittleness, which demonstrates that hardness and strength are not the key factors determining the samples’ fatigue strength; fatigue strength is the combined result of all properties of these materials. It can be seen that the effect of carbide precipitation on the improvement of fatigue strength is not absolute.

**Figure 10:**
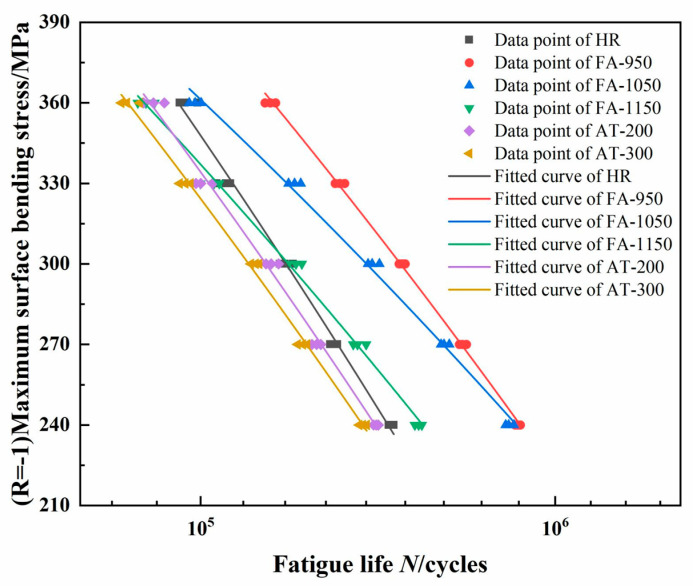
Fatigue curve of composite round steel.

The fatigue life of the samples was tested under different bending stresses, and the fatigue curve plotted and fitted to these data is shown in Figure 10 [27]. S = A + BlgN can be derived from the exponential form of the Basquin equation (e^mS^·N = C), and it shows that the relationship between stress S and life N is semi-logarithmic and linear. The results show that FA-950 exhibits the highest fatigue strength in the relatively high stress range (300 MPa–360 MPa) as it contains carbides that are more dispersed and finer than those in other samples, leading to rare instances of stress concentration. Its fatigue strength is greatly improved due to the combined strengthening of dispersion and fine grains. The carbide particles of FA-1150 are too large and make it more sensitive to high stresses, as they make it easy for stress concentrations and fatigue cracks to form, leading to accelerated cracking, which results in its fatigue strength in the high stress range being lower than that of the HR sample. It can be seen from the fitted fatigue curve that the fatigue limit (N = 10^6^) of FA-950 is the highest (223 MPa). The fatigue limits of AT-200 and AT-300 are 133 MPa and 128 MPa, which are lower than the fatigue limit of HR (135 MPa). We thus know that if precipitated carbides cause a brittle transition in a material, its fatigue strength will decrease despite the strengthening effect of dispersion.

**Figure 11:**
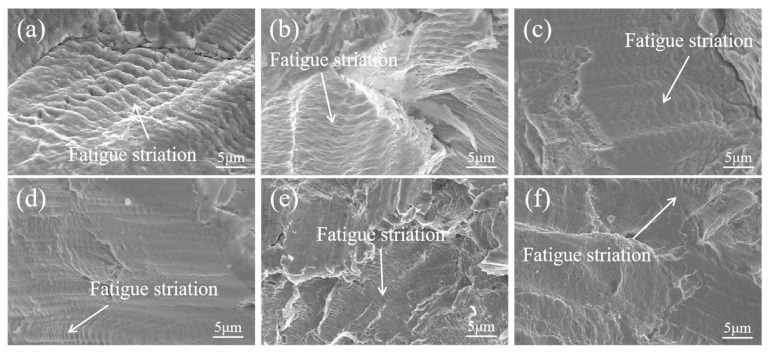
Microscopic morphology of crack propagation region in composite round steel under 360 MPa of bending stress: (**a**) FA-950; (**b**) FA-1050; (**c**) FA-1150; (**d**) HR; (**e**) AT-200; (**f**) AT-300.

**Figure 12:**
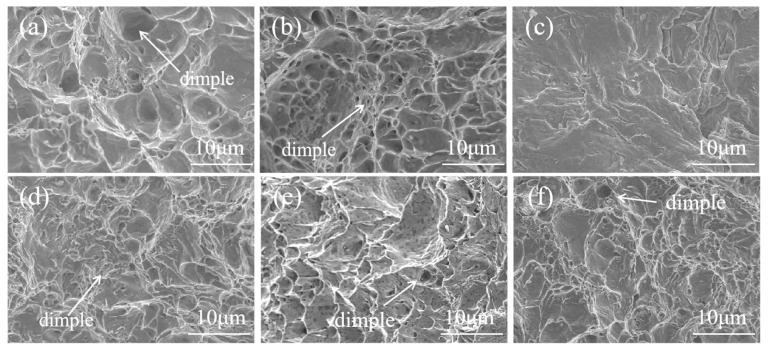
Microscopic morphology of instantaneous fracture region in composite round steel under 360 MPa of bending stress: (**a**) FA-950; (**b**) FA-1050; (**c**) FA-1150; (**d**) HR; (**e**) AT-200; (**f**) AT-300.

Figure 11 shows the area in which crack propagation occurs in composite round steel under 360 MPa of bending stress; it can be seen that the cracks in all samples continue to propagate under cyclic stress, forming fatigue striation bands. The fatigue striations of FA-950 are clearer than most, demonstrating its superior toughness, and the spacing of its fatigue striations is uniform. Figure 12 shows the instantaneous fracture region of the fatigue fracture of composite round steel under 360 MPa of bending stress. It can be seen that FA-950 has larger and deeper dimples compared with the other samples, and its surface has a strong resistance to plastic deformation, even when subjected to higher levels of deformation. With the increase in the full annealing temperature, the diffusion rate of the carbon is accelerated, which leads to the coarsening of carbide particles and hinders the formation of dimples, thus reducing the toughness of the material and its fatigue strength. The small and shallow dimples in the fracture region of AT-200 and AT-300 indicate that their toughness is poor, which is consistent with the tensile results and fatigue strength data.

### 3.3. Corrosion Resistance

**Figure 13:**
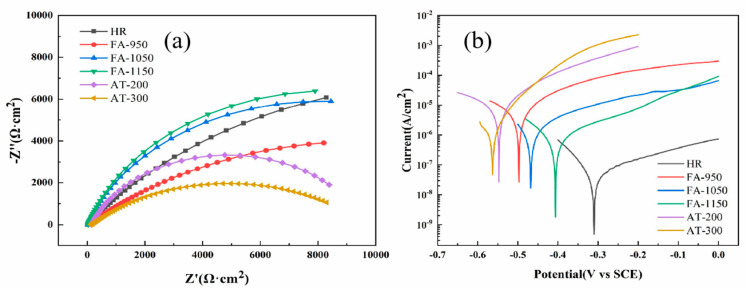
Electrochemical tests: (**a**) EIS; (**b**) Tafel.

In order to evaluate the effect of the heat treatment and chromium carbide on the corrosion resistance of composite round steel, EIS and DL-EPR were carried out and potentiodynamic polarization curves were created. Table 4 shows the electrochemical parameters used. It can be seen from Figure 13 that the whole system is controlled by the charge transfer resistance, with no evidence of mass transfer found in the low-frequency region. The HR sample had the highest charge transfer resistance, with an average R_f_ of 2.6 × 10^4^ Ω·cm^2^. It also has the most positive self-corrosion potential, −0.31 V, and the lowest self-corrosion current density, 5.4 × 10^−8^ A/cm^2^, which indicate that it is the sample that is most resistant to corrosion. This is because carbon cannot diffuse fully in the rapid hot rolling bonding process, meaning that less carbide precipitation occurs, and the chromium is evenly distributed in the matrix, effectively maintaining the stability of the passive film. With the increase in the full annealing temperature, the solid solubility of the chromium carbide in the austenite matrix increases, the amount of precipitates formed decreases significantly, and the ability of the chromium carbide to form a passive film on the surface of the SS decreases. The large amount of chromium carbide that precipitated in FA-950 created a chromium-depleted region in the matrix [28] and led to the formation of Cr_2_O_3_. As chromium is the main component of the passive film, a significant reduction in its continuity and compactness occurs, as well as a significant reduction in the corrosion resistance of the sample [29], leading to rapid corrosion [30]. The average charge transfer resistance R_f_ of this sample is 1.5 × 10^4^Ω·cm^2^; its self-corrosion potential shifts negatively to −0.50 V (vs.SCE), and its self-corrosion current density is 8.2 × 10^−6^ A/cm^2^, which equate to an extremely high corrosion rate. After the aging treatment, more carbides are precipitated, and their ability to form a passive film on the surface is weakened, resulting in a serious decline in corrosion resistance. The charge transfer resistance of AT-300 is the smallest, with an average R_f_ of 9.6 × 10^3^ Ω·cm^2^; additionally, its self-corrosion potential is −0.56 V, and its self-corrosion current density is 7.2 × 10^−6^ A/cm^2^. The results of the EIS and potentiodynamic polarization tests show that the corrosion resistance of composite round steel is seriously affected by the precipitation of a large amount of chromium carbide.

**Figure 14:**
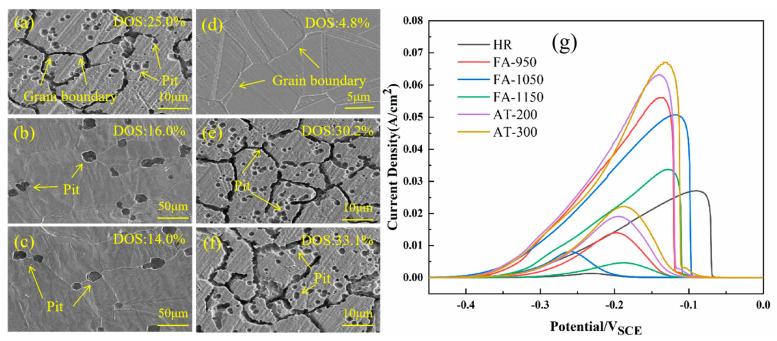
DL-EPR test and corrosion morphology of SS cladding: (**a**) FA-950; (**b**) FA-1050; (**c**) FA-1150; (**d**) HR; (**e**) AT-200; (**f**) AT-300; (**g**) DL-EPR curves.

The DL-EPR curve characteristics of the samples are shown in Figure 14g, while their corrosion morphology is shown in Figure 14a–f. HR exhibited excellent intergranular corrosion resistance (DOS = 4.8%) with a reactivation current peak I_r_ of 1.3 mA/cm^2^, and its passive film was not damaged during the retrace process. It can be observed that HR has almost no corrosion pits distributed along its grain boundary (Figure 14d).

FA-950 has the largest reactivation peak value of 14.0 mA/cm^2^, and this high value reflects that the chromium-depleted region formed by the large number of carbides that were precipitated during annealing has undergone a large amount of corrosion during the retrace [31]; its intergranular corrosion sensitivity is 25.0%. The number and depth of its corrosion pits are significantly larger than those of the other samples, forming a continuous network of corrosion on the grain boundary. The grain boundary is gully-shaped and the large number of corrosion pits in the grain show that FA-950 is subjected to severe intergranular corrosion [32], which confirms that a large number of carbides are precipitated from FA-950, causing the intergranular corrosion phenomenon seen during the DL-EPR test. However, the SS of FA-1050 and FA-1150 still has obvious austenite grain boundaries and fewer corrosion pits, indicating that fewer carbides precipitate at the grain boundaries and within the grains; their SSs still have good intergranular corrosion resistance, which is consistent with results of the DL-EPR test. Intergranular corrosion is increased due to the further intergranular precipitation of carbides after full annealing at 950 °C. With the increase in aging temperature, the peak value of the reactivation current and DOS increase gradually, demonstrating that intergranular sensitization is more serious and causes deeper and more frequent corrosion pits. Among the different samples, AT-300 has the highest reactivation peak value, 22.2 m A/cm^2^, and a DOS value of 33.1%.

## 4. Conclusions

In this study, different carbide precipitation rates were obtained through the use of different full annealing and aging treatment temperatures to create hot-rolled 304/Q235 composite round steel. It was found that carbide has a great impact on the structure and properties of SS/CS metals. Our conclusions are as follows: chromium carbide particles precipitate in a dispersive manner at the grain boundaries, while fine grains and slip lines can significantly weaken the migration of austenite grain boundaries and inhibit the growth of austenite grains due to the pinning effect, resulting in the creation of more austenite grain boundaries. Under the synergistic strengthening effects of dispersion and fine grains, the hardness and strength of composite round steel are greatly improved; the maximum hardness of FA-950 is 402.2 HV, and its yield strength and tensile strength are 270 MPa and 453 MPa, respectively. The stress concentration and the initiation of fatigue cracks are reduced under cyclic stress, with a maximum fatigue limit of 223 MPa (N = 10^6^) for FA-950. However, hardness, yield strength, and tensile strength are not the only factors that determine fatigue strength, as it is also closely related to the brittleness of the material. The growth of chromium carbide may create stress concentration points and accelerate the formation of fatigue cracks, indicating that the precipitation of carbide is not the only factor improving the fatigue strength of these samples. At the same time, the corrosion resistance of composite round steel is reduced with the precipitation of a large amount of chromium carbide. The self-corrosion potential and DOS value of AT-300, which experienced the most serious corrosion, were −0.56 V and 33.1%.

## Figures and Tables

**Table 1:** Chemical composition of experimental steel (wt.%).

Materials	Cr	Ni	C	Mn	Si	P	S	Fe
Q235 carbon steel	-	-	0.22	1.4	0.35	0.045	0.045	balance
304 stainless steel	18.18	8.18	0.057	0.73	0.39	0.026	0.006	balance

**Table 2:** Elemental composition (wt.%).

Spectrogram	Fe	C	Cr	Ni
1	41.32	12.89	40.89	4.90
2	63.49	9.00	15.96	11.55
3	58.03	10.68	22.17	9.12

**Table 3:** Number of fatigue test cycles endured under 360 MPa of bending stress.

Samples	1#	2#	3#	4#	5#	6#	Average
FA-950	116,825	138,762	188,356	126,546	167,347	176,548	152,397
FA-1050	103,321	104,542	109,843	85,321	81,569	96,626	96,870
FA-1150	73,780	71,543	69,423	75,483	73,875	71,952	72,676
HR	85,467	95,782	102,895	88,935	91,654	82,856	91,265
AT-200	62,434	75,943	93,654	71,764	68,534	80,930	75,543
AT-300	59,789	66,198	89,351	63,764	65,234	70,223	69,093

**Table 4:** Electrochemical parameters of stainless-steel cladding.

Sample	FA-950	F5-1050	FA-1150	HR	AT-200	AT-300
E_corr_(V)	−0.50	−0.47	−0.41	−0.31	−0.55	−0.56
I_corr_(A/cm^2^)	8.2 × 10^−6^	1.2 × 10^−6^	7.4 × 10^−7^	5.4 × 10^−8^	8.4 × 10^−6^	7.2 × 10^−6^
R_f_(Ω·cm^2^)	1.5 × 10^4^	1.6 × 10^4^	1.7 × 10^4^	2.6 × 10^4^	1.3 × 10^4^	9.6 × 10^3^
I_r_(mA/cm^2^)	14.0	8.1	4.7	1.3	19.1	22.2
I_a_(mA/cm^2^)	56.1	50.7	33.7	27.1	63.2	67.0
DOS(I_r_/I_a_) × 100%	25.0	16.0	14.0	4.8	30.2	33.1

## Data Availability

The original contributions presented in the study are included in the article, further inquiries can be directed to the corresponding author.

## References

[1] Mutlu I.H., Emre M.C., Kaya A.O. (2023). A comparison of the corrosion resistance of galvanized low steel with solgel method coated ZrO2, ZrO2+Polymer coating. Kuwait J. Sci..

[2] Lu J.Z., Deng W.W., Luo K.Y., Wu L.J., Lu H.F. (2017). Surface EBSD analysis and strengthening mechanism of AISI304 stainless steel subjected to massive LSP treatment with different pulse energies. Mater. Charact..

[3] Xu W., Yang Y., Dai C., Xie J. (2020). Optimization of spinning parameters of 20/316L bimetal composite tube based on orthogonal test. Sci. Eng. Compos. Mater..

[4] Wang S., Zhao G., Li Y., Li J., Song Y. (2019). Composite Plate Rolling Technology of 304/Q345R Based on a Corrugated Interface. Materials.

[5] Wang J., Zhao F., Xie G., Hou Y., Wang R., Liu X. (2021). Rolling deformation behaviour and interface evaluation of Cu-Al bimetallic composite plates fabricated by horizontal continuous composite casting. J. Mater. Process. Technol..

[6] Jiang W., Fan Z., Li G., Li C. (2016). Effects of zinc coating on interfacial microstructures and mechanical properties of aluminum/steel bimetallic composites. J. Alloys Compd..

[7] Abdelkader W.B., Bahloul R., Arfa H. (2020). Numerical investigation of the influence of some parameters in SPIF process on the forming forces and thickness distributions of a bimetallic sheet CP-titanium/low-carbon steel compared to an individual layer. Procedia Manuf..

[8] Li Z., Xie H., Jia F., Lu Y., Yuan X., Jiao S., Jiang Z. (2020). Study on Deformation Characteristics and Microstructure Evolution of 2205/AH36 Bimetal Composite in a Novel Hot Forming Process. Metals.

[9] Li H., Zhang L., Zhang B., Zhang Q. (2020). Effect of heat treatment on the microstructure and corrosion resistance of stainless/carbon steel bimetal plate. Adv. Mater. Sci. Eng..

[10] Huang Q., Yang X., Ma L., Zhou C., Guang M., Li H. (2014). Interface-correlated characteristics of stainless steel/carbon steel plate fabricated by AAWIV and hot rolling. J. Iron Steel Res. Int..

[11] Kaya Y., Kahraman N., Durgutlu A., Gülenç B. (2017). Investigation of the microstructural, mechanical and corrosion properties of grade a ship steel-duplex stainless steel composites produced via explosive welding. Metall. Mater. Trans. A.

[12] Su H., Luo X., Chai F., Shen J., Sun X., Lu F. (2015). Manufacturing technology and application trends of titanium clad steel plates. J. Iron Steel Res. Int..

[13] Tan J., Gao Z., Ren S., Xu Q., Wang K., Zeng Q. (2024). Improving the microstructures and mechanical properties of U71Mn rail steel liner friction welded joint by normalizing treatment. Mater. Today Commun..

[14] Sarkar A., Modak P., Mandal A., Chakrabarti D., Karmakar A. (2024). Correlation between microstructure and tensile properties of low-carbon steel processed via different thermomechanical routes. J. Mater. Eng. Perform..

[15] Wang H., Wang A., Li C., Yu X., Xie J., Liu C. (2022). Effect of secondary-phase precipitation on mechanical properties and corrosion resistance of 00cr27ni7mo5n hyper-duplex stainless steel during solution treatment. Materials.

[16] Wang J., Shi W., Xiang S., Ballinger R.G. (2021). Study of the corrosion behaviour of sensitized 904L austenitic stainless steel in Cl-solution. Corros. Sci..

[17] Ding B., Zhao Y., Huang Z., Cai L., Wang N. (2020). Tensile bearing capacity for bolted spherical joints with different screwing depths of high-strength bolts. Eng. Struct..

[18] Dhib Z., Guermazi N., Gaspérini M., Haddar N. (2016). Cladding of low-carbon steel to austenitic stainless steel by hot-roll bonding: Microstructure and mechanical properties before and after welding. Mater. Sci. Eng. A.

[19] Yang L., Wang Y., Guan J., Zhang Y., Shi Y. (2015). Bearing strength of stainless steel bolted connections. Adv. Struct. Eng..

[20] Salman O.O., Gammer C., Chaubey A.K., Eckert J., Scudino S. (2019). Effect of heat treatment on microstructure and mechanical properties of 316L steel synthesized by selective laser melting. Mater. Sci. Eng. A.

[21] Zhao B., Xie L., Song J., Ren J., Wang B., Zhang S. (2020). Fatigue life prediction of aero-engine compressor disk based on a new stress field intensity approach. Int. J. Mech. Sci..

[22] Li G., Wang S., He J., Wu K., Zhou C. (2019). Compilation of load spectrum of machining center spindle and application in fatigue life prediction. J. Mech. Sci. Technol..

[23] Yang Y., Jiang Z., Chen Y., Liu X., Sun J., Wang W. (2022). Interfacial microstructure and strengthening mechanism of stainless steel/carbon steel laminated composite fabricated by liquid-solid bonding and hot rolling. Mater. Charact..

[24] Qi X., Huan P., Wang X., Shen X., Liu Z., Di H. (2022). Effect of microstructure homogeneity on the impact fracture mechanism of X100 pipeline steel laser–MAG hybrid welds with an alternating magnetic field. Mater. Sci. Eng. A.

[25] Wu B., Guo K., Yang X., Gao Y., Jin Y., Gao Y., Wang Q., Zhang F. (2022). Effect of carbon content of substrate on the microstructure changes and tensile behavior of clad layer of stainless steel composites. Mater. Sci. Eng. A.

[26] Huang X., Wang D., Yang Y. (2015). Effect of precipitation on intergranular corrosion resistance of 430 ferritic stainless steel. J. Iron Steel Res. Int..

[27] Zhou Z., Ding Y. (2023). A study on the fatigue performance and corrosion resistance of 304/45 bimetallic composite bolts. Materials.

[28] Kina A.Y., Souza V.M.D., Tavares S.S.M., Souza J.A.D., de Abreu H.F.G. (2008). Influence of heat treatments on the intergranular corrosion resistance of the AISI 347 cast and weld metal for high temperature services. J. Mater. Process. Technol..

[29] Kaneko K., Fukunaga T., Yamada K., Nakada N., Kikuchi M., Saghi Z., Barnard J.S., Midgley P.A. (2011). Formation of M23C6-type precipitates and chromium-depleted zones in austenite stainless steel. Scr. Mater..

[30] Mohammadi F., Nickchi T., Attar M.M., Alfantazi A. (2011). EIS study of potentiostatically formed passive film on 304 stainless steel. Electrochim. Acta.

[31] Hang P., Zhao B., Zhou J., Ding Y. (2023). Effect of Heat Treatment on Crevice Corrosion Behavior of 304 Stainless Steel Clad Plate in Seawater Environment. Materials.

[32] Li L., Zhao B., Chen Y., Ding Y. (2023). Effect of Heat Treatment on Microstructure and Properties Evolution of Stainless Steel Cladding Plate. Materials.

